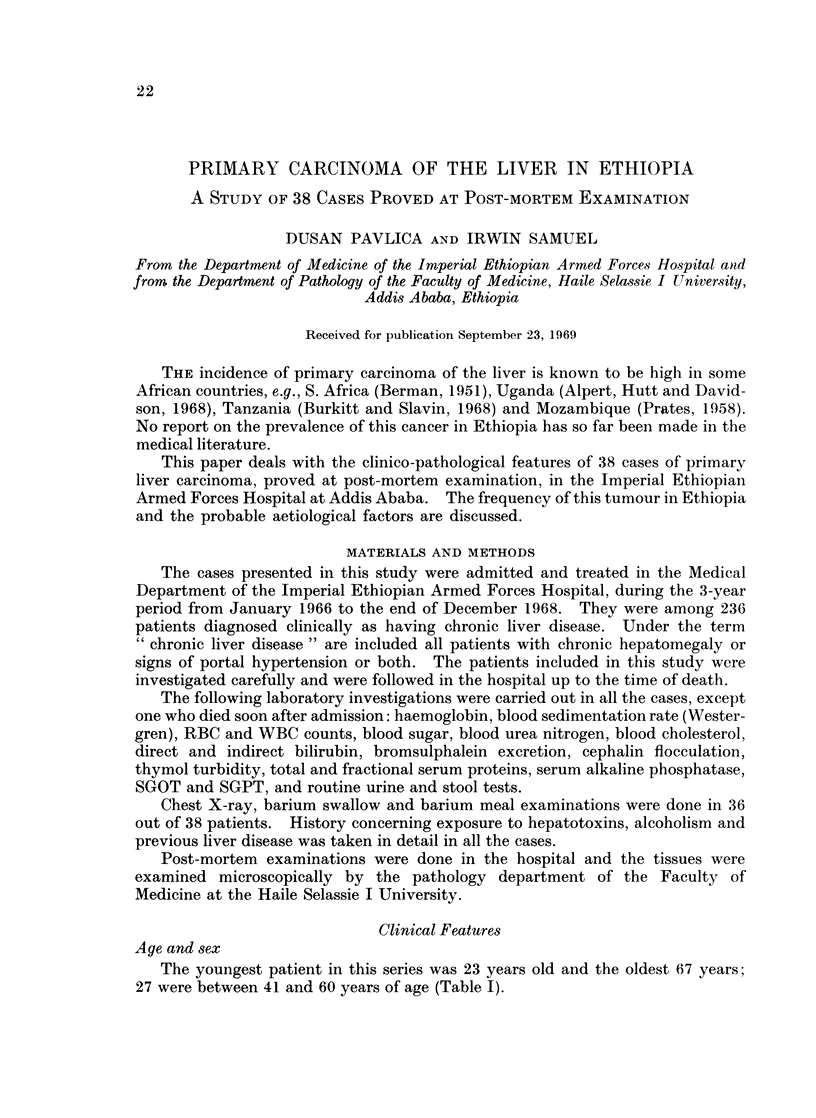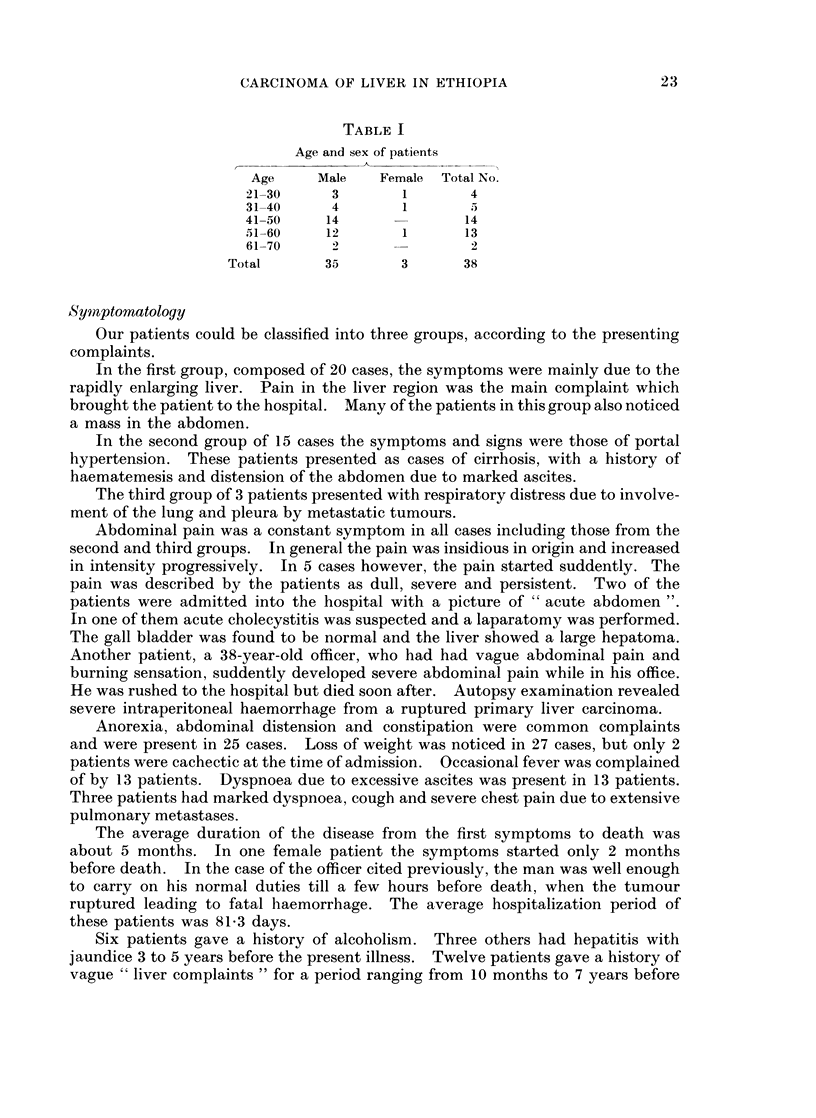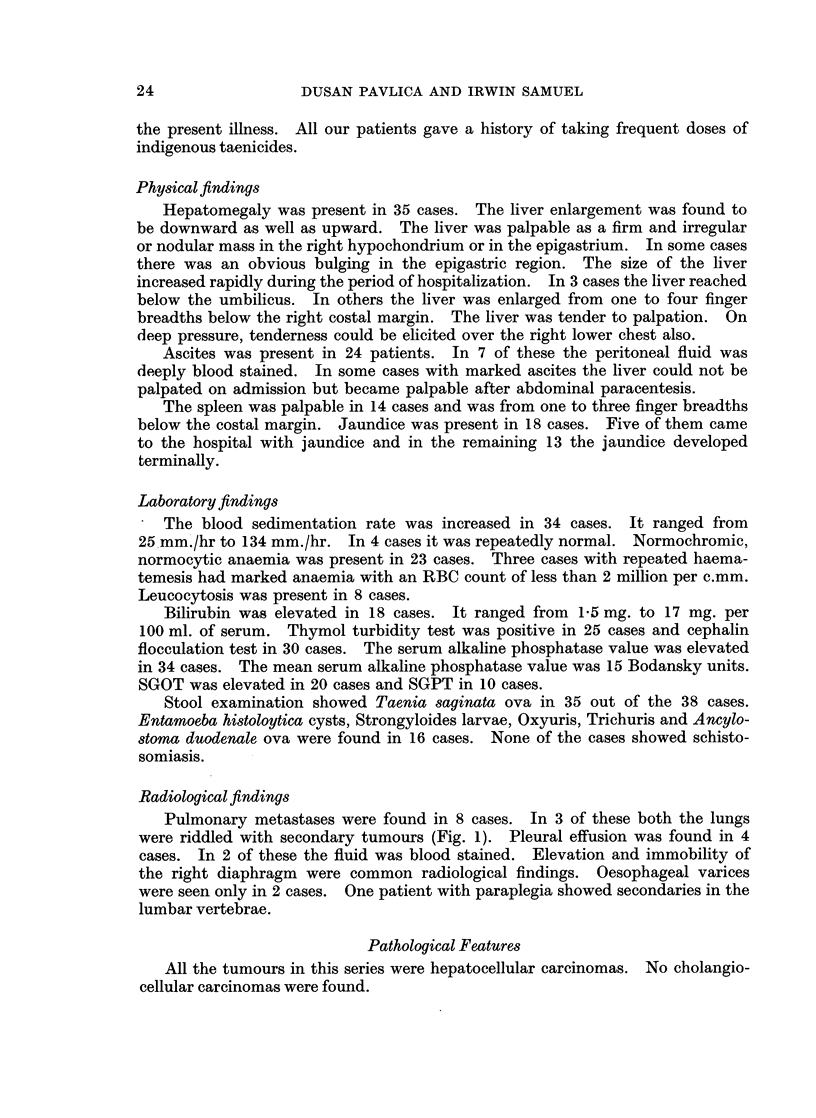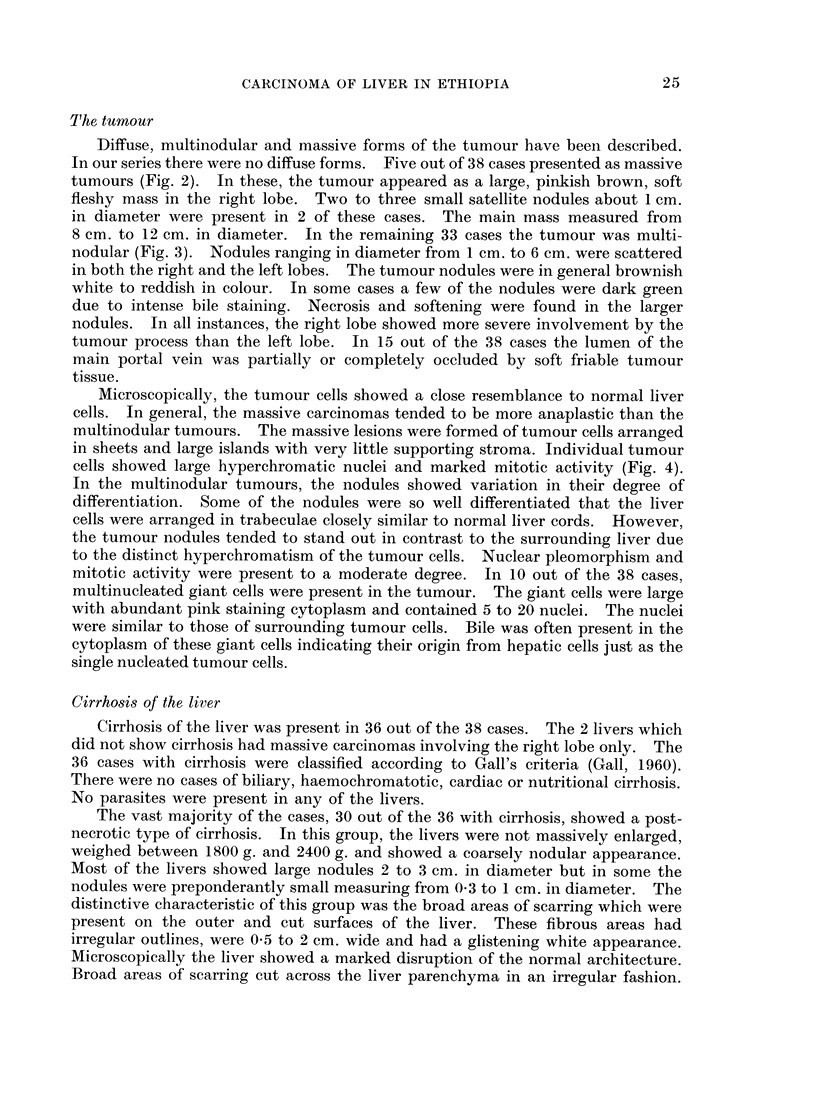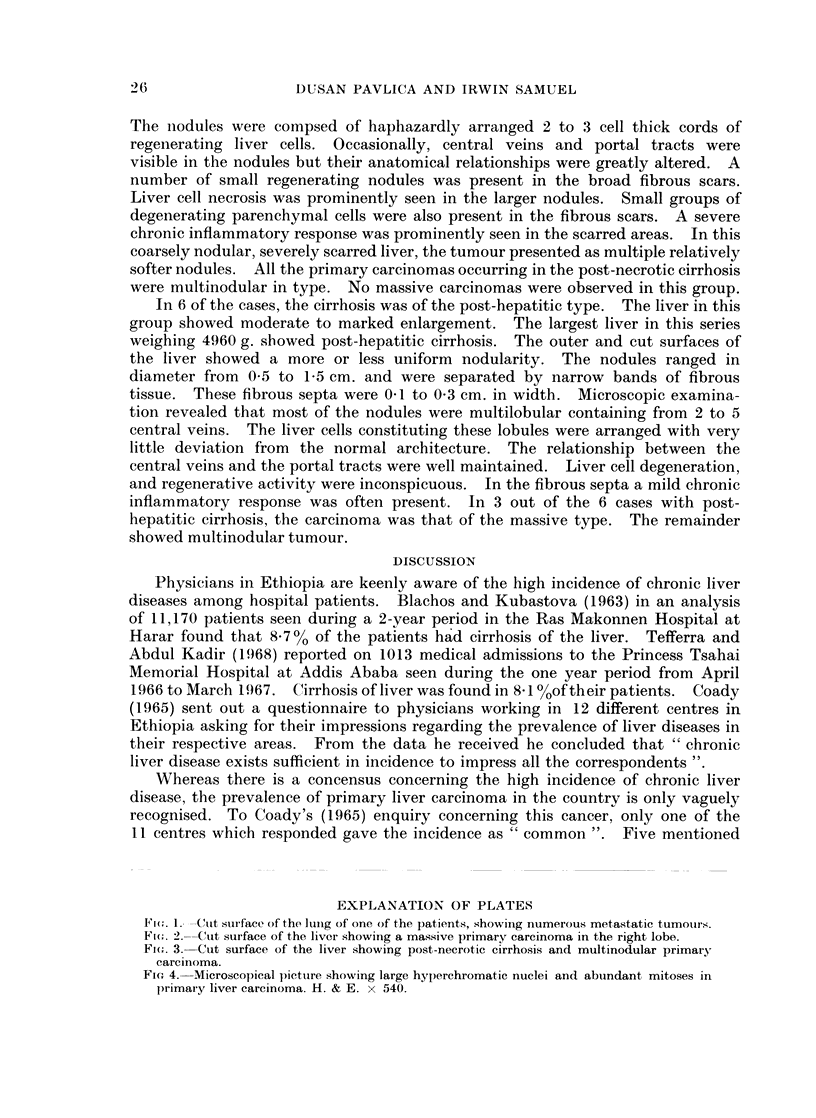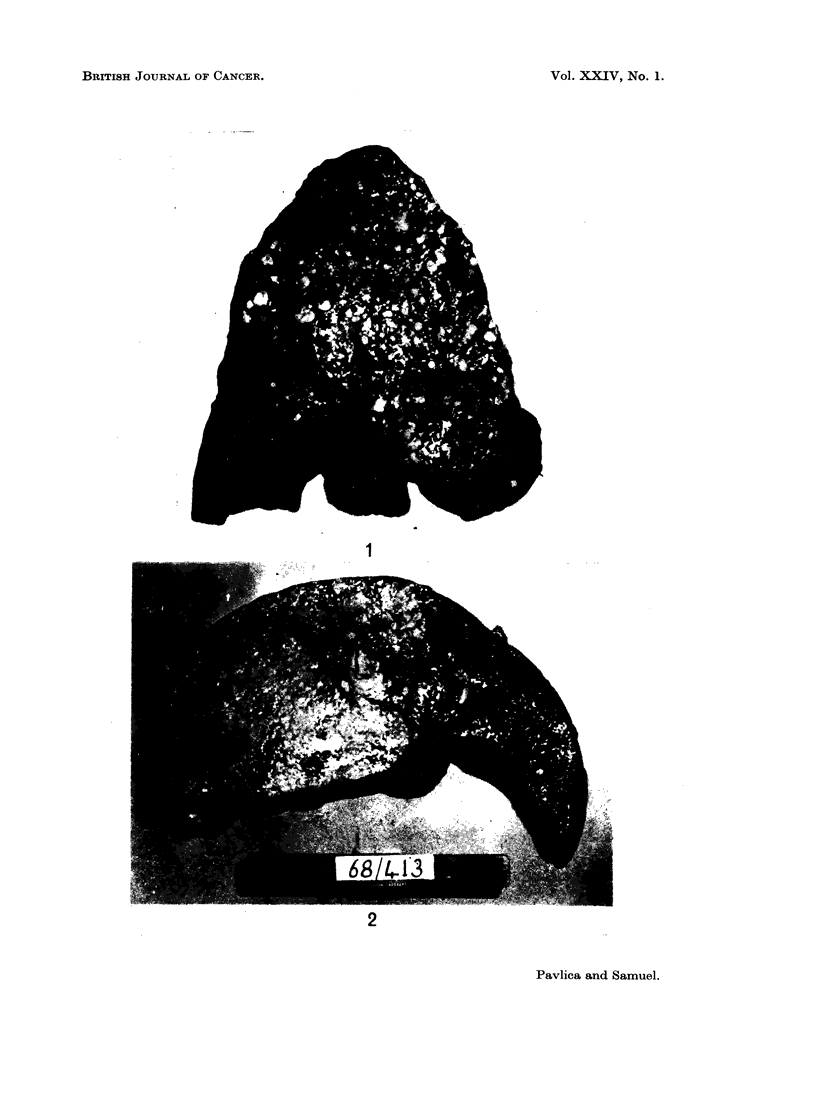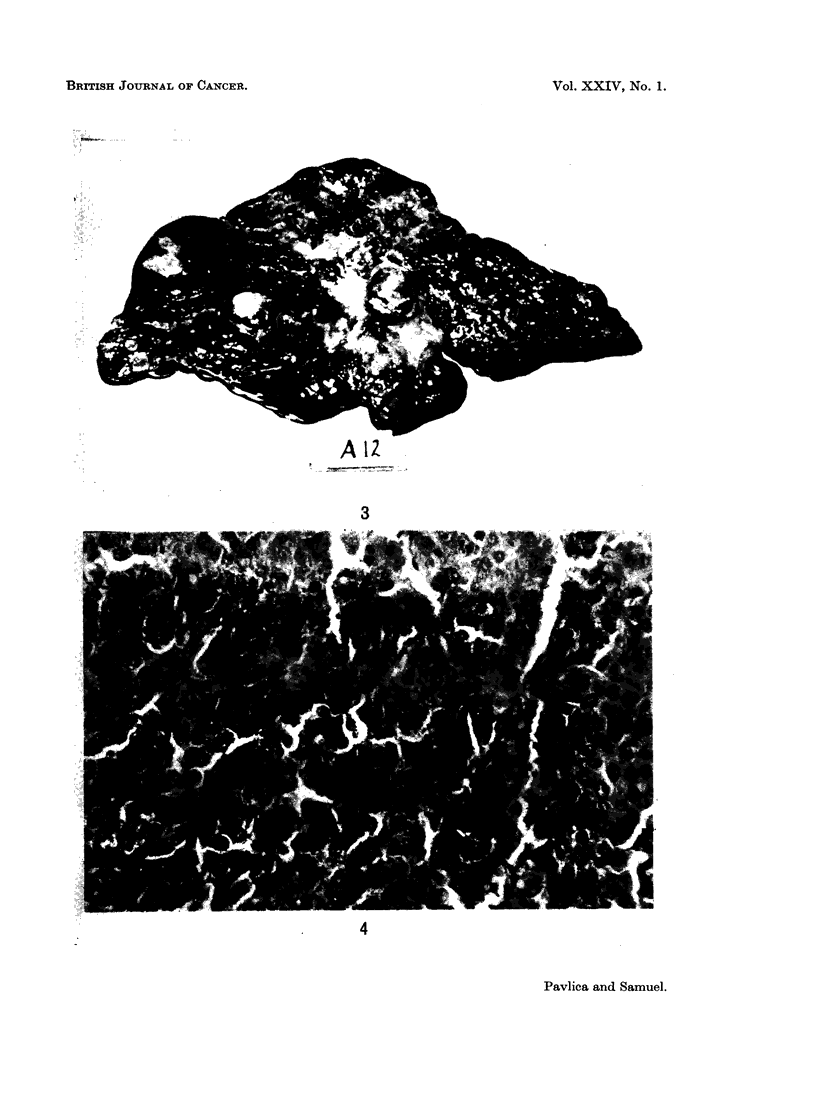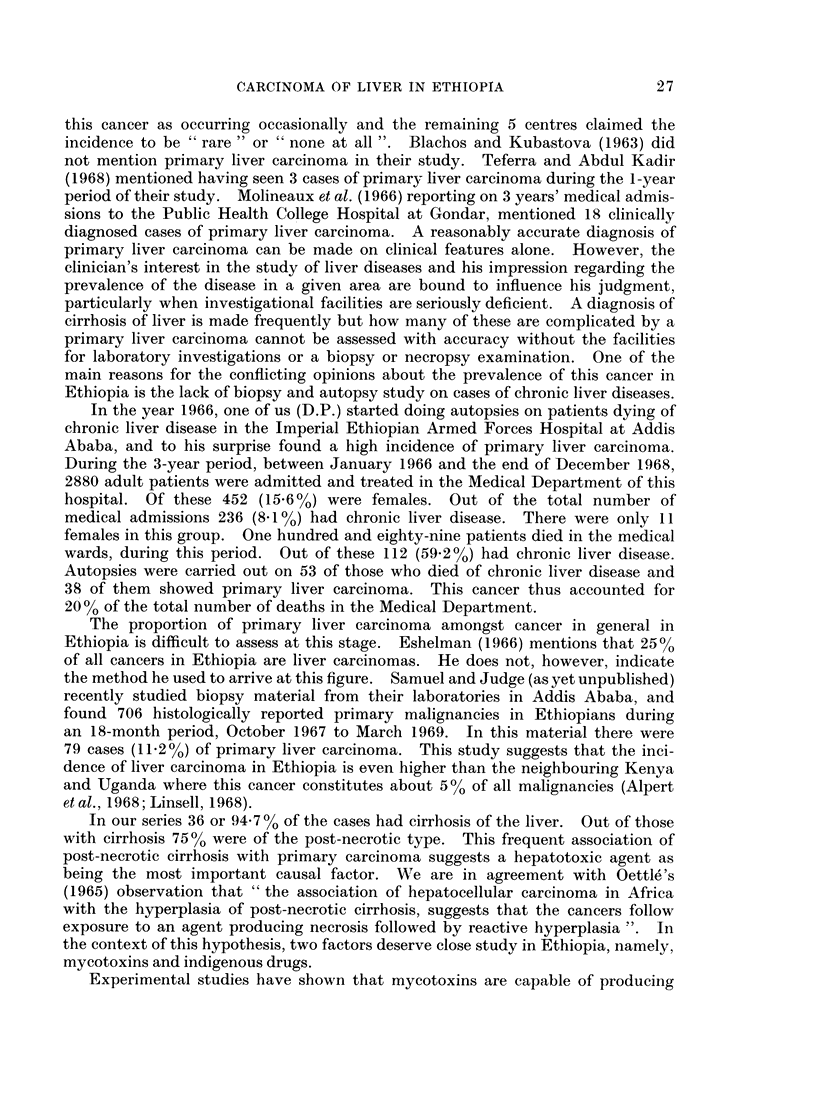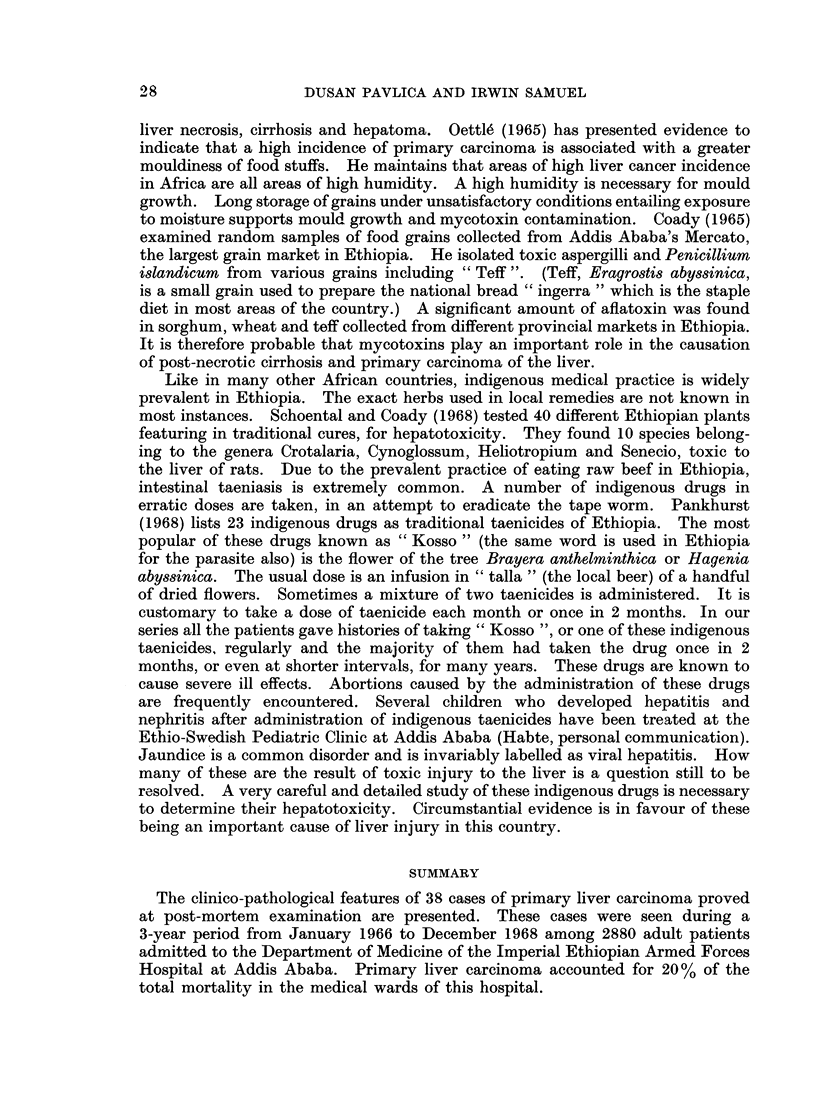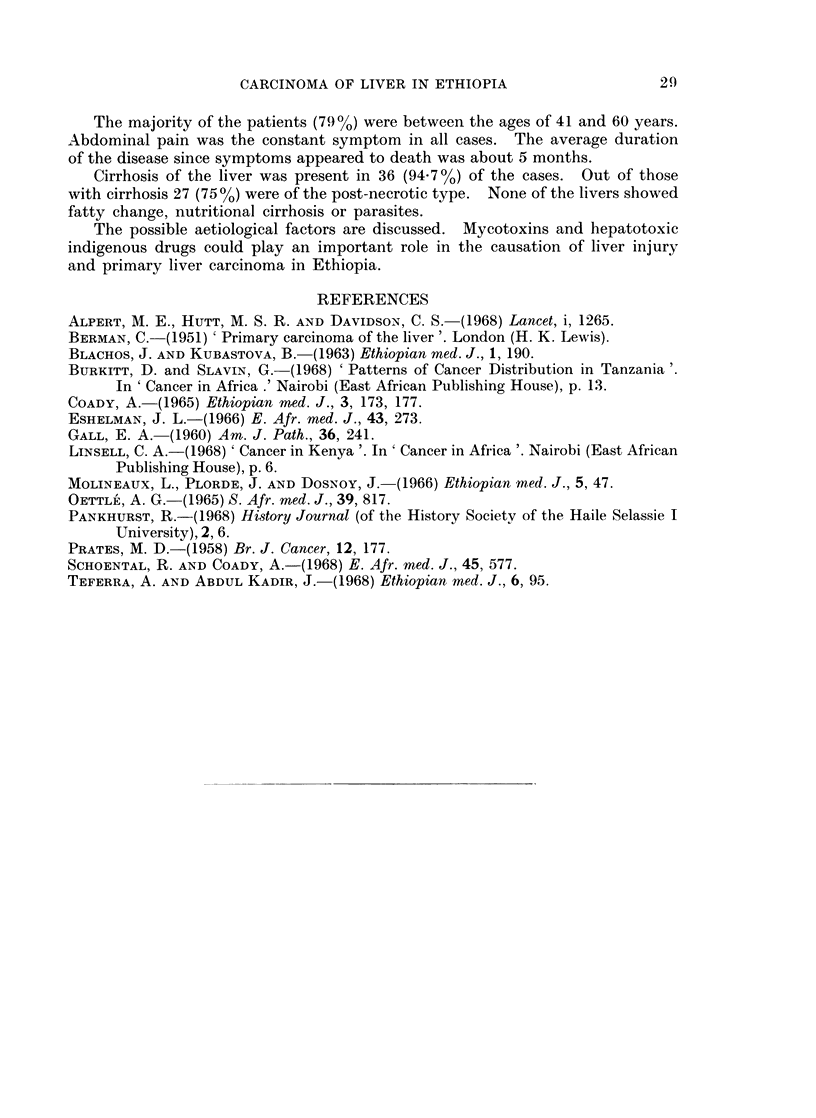# Primary carcinoma of the liver in Ethiopia. A study of 38 cases proved at post-mortem examination.

**DOI:** 10.1038/bjc.1970.4

**Published:** 1970-03

**Authors:** D. Pavlica, I. Samuel

## Abstract

**Images:**


### PRIMARY CARCINOMA OF THE LIVER IN ETHIOPIA

### A Study of 38 Cases Proved at Post-mortem Examination

DUSAN PAVLICA and IRWIN SAMUEL

*From the Department of Medicine of the Imperial Ethiopian Armed Forces Hospital and from the Department of Pathology of the Faculty of Medicine, Haile Selassie I University, Addis Ababa, Ethiopia*

Received for publication September 23, 1969

THE incidence of primary carcinoma of the liver is known to be high in some African countries, *e.g.*, S. Africa (Berman, 1951), Uganda (Alpert, Hutt and Davidson, 1968), Tanzania (Burkitt and Slavin, 1968) and Mozambique (Prates, 1958). No report on the prevalence of this cancer in Ethiopia has so far been made in the medical literature.

This paper deals with the clinico-pathological features of 38 cases of primary liver carcinoma, proved at post-mortem examination, in the Imperial Ethiopian Armed Forces Hospital at Addis Ababa. The frequency of this tumour in Ethiopia and the probable aetiological factors are discussed.

### MATERIALS AND METHODS

The cases presented in this study were admitted and treated in the Medical Department of the Imperial Ethiopian Armed Forces Hospital, during the 3-year period from January 1966 to the end of December 1968. They were among 236 patients diagnosed clinically as having chronic liver disease. Under the term " chronic liver disease " are included all patients with chronic hepatomegaly or signs of portal hypertension or both. The patients included in this study were investigated carefully and were followed in the hospital up to the time of death.

The following laboratory investigations were carried out in all the cases, except one who died soon after admission: haemoglobin, blood sedimentation rate (Westergren), RBC and WBC counts, blood sugar, blood urea nitrogen, blood cholesterol, direct and indirect bilirubin, bromsulphalein excretion, cephalin flocculation, thymol turbidity, total and fractional serum proteins, serum alkaline phosphatase, SGOT and SGPT, and routine urine and stool tests.

Chest X-ray, barium swallow and barium meal examinations were done in 36 out of 38 patients. History concerning exposure to hepatotoxins, alcoholism and previous liver disease was taken in detail in all the cases.

Post-mortem examinations were done in the hospital and the tissues were examined microscopically by the pathology department of the Faculty of Medicine at the Haile Selassie I University.

### *Clinical Features*

#### *Age and sex*

The youngest patient in this series was 23 years old and the oldest 67 years; 27 were between 41 and 60 years of age (Table I).

TABLE I

Age and sex of patients

| Age | Male | Female | Total No. |
|---|---|---|---|
| 21–30 | 3 | 1 | 4 |
| 31–40 | 4 | 1 | 5 |
| 41–50 | 14 | — | 14 |
| 51–60 | 12 | 1 | 13 |
| 61–70 | 2 | — | 2 |
| Total | 35 | 3 | 38 |

*Symptomatology*

Our patients could be classified into three groups, according to the presenting complaints.

In the first group, composed of 20 cases, the symptoms were mainly due to the rapidly enlarging liver. Pain in the liver region was the main complaint which brought the patient to the hospital. Many of the patients in this group also noticed a mass in the abdomen.

In the second group of 15 cases the symptoms and signs were those of portal hypertension. These patients presented as cases of cirrhosis, with a history of haematemesis and distension of the abdomen due to marked ascites.

The third group of 3 patients presented with respiratory distress due to involvement of the lung and pleura by metastatic tumours.

Abdominal pain was a constant symptom in all cases including those from the second and third groups. In general the pain was insidious in origin and increased in intensity progressively. In 5 cases however, the pain started suddently. The pain was described by the patients as dull, severe and persistent. Two of the patients were admitted into the hospital with a picture of " acute abdomen ". In one of them acute cholecystitis was suspected and a laparatomy was performed. The gall bladder was found to be normal and the liver showed a large hepatoma. Another patient, a 38-year-old officer, who had had vague abdominal pain and burning sensation, suddently developed severe abdominal pain while in his office. He was rushed to the hospital but died soon after. Autopsy examination revealed severe intraperitoneal haemorrhage from a ruptured primary liver carcinoma.

Anorexia, abdominal distension and constipation were common complaints and were present in 25 cases. Loss of weight was noticed in 27 cases, but only 2 patients were cachectic at the time of admission. Occasional fever was complained of by 13 patients. Dyspnoea due to excessive ascites was present in 13 patients. Three patients had marked dyspnoea, cough and severe chest pain due to extensive pulmonary metastases.

The average duration of the disease from the first symptoms to death was about 5 months. In one female patient the symptoms started only 2 months before death. In the case of the officer cited previously, the man was well enough to carry on his normal duties till a few hours before death, when the tumour ruptured leading to fatal haemorrhage. The average hospitalization period of these patients was 81·3 days.

Six patients gave a history of alcoholism. Three others had hepatitis with jaundice 3 to 5 years before the present illness. Twelve patients gave a history of vague " liver complaints " for a period ranging from 10 months to 7 years before the present illness. All our patients gave a history of taking frequent doses of indigenous taenicides.

*Physical findings*

Hepatomegaly was present in 35 cases. The liver enlargement was found to be downward as well as upward. The liver was palpable as a firm and irregular or nodular mass in the right hypochondrium or in the epigastrium. In some cases there was an obvious bulging in the epigastric region. The size of the liver increased rapidly during the period of hospitalization. In 3 cases the liver reached below the umbilicus. In others the liver was enlarged from one to four finger breadths below the right costal margin. The liver was tender to palpation. On deep pressure, tenderness could be elicited over the right lower chest also.

Ascites was present in 24 patients. In 7 of these the peritoneal fluid was deeply blood stained. In some cases with marked ascites the liver could not be palpated on admission but became palpable after abdominal paracentesis.

The spleen was palpable in 14 cases and was from one to three finger breadths below the costal margin. Jaundice was present in 18 cases. Five of them came to the hospital with jaundice and in the remaining 13 the jaundice developed terminally.

*Laboratory findings*

The blood sedimentation rate was increased in 34 cases. It ranged from 25 mm./hr to 134 mm./hr. In 4 cases it was repeatedly normal. Normochromic, normocytic anaemia was present in 23 cases. Three cases with repeated haematemesis had marked anaemia with an RBC count of less than 2 million per c.mm. Leucocytosis was present in 8 cases.

Bilirubin was elevated in 18 cases. It ranged from 1·5 mg. to 17 mg. per 100 ml. of serum. Thymol turbidity test was positive in 25 cases and cephalin flocculation test in 30 cases. The serum alkaline phosphatase value was elevated in 34 cases. The mean serum alkaline phosphatase value was 15 Bodansky units. SGOT was elevated in 20 cases and SGPT in 10 cases.

Stool examination showed *Taenia saginata* ova in 35 out of the 38 cases. *Entamoeba histoloytica* cysts, Strongyloides larvae, Oxyuris, Trichuris and *Ancylostoma duodenale* ova were found in 16 cases. None of the cases showed schistosomiasis.

*Radiological findings*

Pulmonary metastases were found in 8 cases. In 3 of these both the lungs were riddled with secondary tumours (Fig. 1). Pleural effusion was found in 4 cases. In 2 of these the fluid was blood stained. Elevation and immobility of the right diaphragm were common radiological findings. Oesophageal varices were seen only in 2 cases. One patient with paraplegia showed secondaries in the lumbar vertebrae.

### *Pathological Features*

All the tumours in this series were hepatocellular carcinomas. No cholangiocellular carcinomas were found.

*The tumour*

Diffuse, multinodular and massive forms of the tumour have been described. In our series there were no diffuse forms. Five out of 38 cases presented as massive tumours (Fig. 2). In these, the tumour appeared as a large, pinkish brown, soft fleshy mass in the right lobe. Two to three small satellite nodules about 1 cm. in diameter were present in 2 of these cases. The main mass measured from 8 cm. to 12 cm. in diameter. In the remaining 33 cases the tumour was multinodular (Fig. 3). Nodules ranging in diameter from 1 cm. to 6 cm. were scattered in both the right and the left lobes. The tumour nodules were in general brownish white to reddish in colour. In some cases a few of the nodules were dark green due to intense bile staining. Necrosis and softening were found in the larger nodules. In all instances, the right lobe showed more severe involvement by the tumour process than the left lobe. In 15 out of the 38 cases the lumen of the main portal vein was partially or completely occluded by soft friable tumour tissue.

Microscopically, the tumour cells showed a close resemblance to normal liver cells. In general, the massive carcinomas tended to be more anaplastic than the multinodular tumours. The massive lesions were formed of tumour cells arranged in sheets and large islands with very little supporting stroma. Individual tumour cells showed large hyperchromatic nuclei and marked mitotic activity (Fig. 4). In the multinodular tumours, the nodules showed variation in their degree of differentiation. Some of the nodules were so well differentiated that the liver cells were arranged in trabeculae closely similar to normal liver cords. However, the tumour nodules tended to stand out in contrast to the surrounding liver due to the distinct hyperchromatism of the tumour cells. Nuclear pleomorphism and mitotic activity were present to a moderate degree. In 10 out of the 38 cases, multinucleated giant cells were present in the tumour. The giant cells were large with abundant pink staining cytoplasm and contained 5 to 20 nuclei. The nuclei were similar to those of surrounding tumour cells. Bile was often present in the cytoplasm of these giant cells indicating their origin from hepatic cells just as the single nucleated tumour cells.

*Cirrhosis of the liver*

Cirrhosis of the liver was present in 36 out of the 38 cases. The 2 livers which did not show cirrhosis had massive carcinomas involving the right lobe only. The 36 cases with cirrhosis were classified according to Gall's criteria (Gall, 1960). There were no cases of biliary, haemochromatotic, cardiac or nutritional cirrhosis. No parasites were present in any of the livers.

The vast majority of the cases, 30 out of the 36 with cirrhosis, showed a post-necrotic type of cirrhosis. In this group, the livers were not massively enlarged, weighed between 1800 g. and 2400 g. and showed a coarsely nodular appearance. Most of the livers showed large nodules 2 to 3 cm. in diameter but in some the nodules were preponderantly small measuring from 0·3 to 1 cm. in diameter. The distinctive characteristic of this group was the broad areas of scarring which were present on the outer and cut surfaces of the liver. These fibrous areas had irregular outlines, were 0·5 to 2 cm. wide and had a glistening white appearance. Microscopically the liver showed a marked disruption of the normal architecture. Broad areas of scarring cut across the liver parenchyma in an irregular fashion.

The nodules were compsed of haphazardly arranged 2 to 3 cell thick cords of regenerating liver cells. Occasionally, central veins and portal tracts were visible in the nodules but their anatomical relationships were greatly altered. A number of small regenerating nodules was present in the broad fibrous scars. Liver cell necrosis was prominently seen in the larger nodules. Small groups of degenerating parenchymal cells were also present in the fibrous scars. A severe chronic inflammatory response was prominently seen in the scarred areas. In this coarsely nodular, severely scarred liver, the tumour presented as multiple relatively softer nodules. All the primary carcinomas occurring in the post-necrotic cirrhosis were multinodular in type. No massive carcinomas were observed in this group.

In 6 of the cases, the cirrhosis was of the post-hepatitic type. The liver in this group showed moderate to marked enlargement. The largest liver in this series weighing 4960 g. showed post-hepatitic cirrhosis. The outer and cut surfaces of the liver showed a more or less uniform nodularity. The nodules ranged in diameter from 0·5 to 1·5 cm. and were separated by narrow bands of fibrous tissue. These fibrous septa were 0·1 to 0·3 cm. in width. Microscopic examination revealed that most of the nodules were multilobular containing from 2 to 5 central veins. The liver cells constituting these lobules were arranged with very little deviation from the normal architecture. The relationship between the central veins and the portal tracts were well maintained. Liver cell degeneration, and regenerative activity were inconspicuous. In the fibrous septa a mild chronic inflammatory response was often present. In 3 out of the 6 cases with post-hepatitic cirrhosis, the carcinoma was that of the massive type. The remainder showed multinodular tumour.

### DISCUSSION

Physicians in Ethiopia are keenly aware of the high incidence of chronic liver diseases among hospital patients. Blachos and Kubastova (1963) in an analysis of 11,170 patients seen during a 2-year period in the Ras Makonnen Hospital at Harar found that 8·7% of the patients had cirrhosis of the liver. Tefferra and Abdul Kadir (1968) reported on 1013 medical admissions to the Princess Tsahai Memorial Hospital at Addis Ababa seen during the one year period from April 1966 to March 1967. Cirrhosis of liver was found in 8·1 %of their patients. Coady (1965) sent out a questionnaire to physicians working in 12 different centres in Ethiopia asking for their impressions regarding the prevalence of liver diseases in their respective areas. From the data he received he concluded that " chronic liver disease exists sufficient in incidence to impress all the correspondents ".

Whereas there is a concensus concerning the high incidence of chronic liver disease, the prevalence of primary liver carcinoma in the country is only vaguely recognised. To Coady's (1965) enquiry concerning this cancer, only one of the 11 centres which responded gave the incidence as " common ". Five mentioned

### EXPLANATION OF PLATES

FIG. 1.—Cut surface of the lung of one of the patients, showing numerous metastatic tumours.
FIG. 2.—Cut surface of the liver showing a massive primary carcinoma in the right lobe.
FIG. 3.—Cut surface of the liver showing post-necrotic cirrhosis and multinodular primary carcinoma.
FIG 4.—Microscopical picture showing large hyperchromatic nuclei and abundant mitoses in primary liver carcinoma. H. & E. × 540.

1

2

3

4

Pavlica and Samuel.

this cancer as occurring occasionally and the remaining 5 centres claimed the incidence to be " rare " or " none at all ". Blachos and Kubastova (1963) did not mention primary liver carcinoma in their study. Teferra and Abdul Kadir (1968) mentioned having seen 3 cases of primary liver carcinoma during the 1-year period of their study. Molineaux *et al.* (1966) reporting on 3 years' medical admissions to the Public Health College Hospital at Gondar, mentioned 18 clinically diagnosed cases of primary liver carcinoma. A reasonably accurate diagnosis of primary liver carcinoma can be made on clinical features alone. However, the clinician's interest in the study of liver diseases and his impression regarding the prevalence of the disease in a given area are bound to influence his judgment, particularly when investigational facilities are seriously deficient. A diagnosis of cirrhosis of liver is made frequently but how many of these are complicated by a primary liver carcinoma cannot be assessed with accuracy without the facilities for laboratory investigations or a biopsy or necropsy examination. One of the main reasons for the conflicting opinions about the prevalence of this cancer in Ethiopia is the lack of biopsy and autopsy study on cases of chronic liver diseases.

In the year 1966, one of us (D.P.) started doing autopsies on patients dying of chronic liver disease in the Imperial Ethiopian Armed Forces Hospital at Addis Ababa, and to his surprise found a high incidence of primary liver carcinoma. During the 3-year period, between January 1966 and the end of December 1968, 2880 adult patients were admitted and treated in the Medical Department of this hospital. Of these 452 (15·6%) were females. Out of the total number of medical admissions 236 (8·1%) had chronic liver disease. There were only 11 females in this group. One hundred and eighty-nine patients died in the medical wards, during this period. Out of these 112 (59·2%) had chronic liver disease. Autopsies were carried out on 53 of those who died of chronic liver disease and 38 of them showed primary liver carcinoma. This cancer thus accounted for 20% of the total number of deaths in the Medical Department.

The proportion of primary liver carcinoma amongst cancer in general in Ethiopia is difficult to assess at this stage. Eshelman (1966) mentions that 25% of all cancers in Ethiopia are liver carcinomas. He does not, however, indicate the method he used to arrive at this figure. Samuel and Judge (as yet unpublished) recently studied biopsy material from their laboratories in Addis Ababa, and found 706 histologically reported primary malignancies in Ethiopians during an 18-month period, October 1967 to March 1969. In this material there were 79 cases (11·2%) of primary liver carcinoma. This study suggests that the incidence of liver carcinoma in Ethiopia is even higher than the neighbouring Kenya and Uganda where this cancer constitutes about 5% of all malignancies (Alpert *et al.*, 1968; Linsell, 1968).

In our series 36 or 94·7% of the cases had cirrhosis of the liver. Out of those with cirrhosis 75% were of the post-necrotic type. This frequent association of post-necrotic cirrhosis with primary carcinoma suggests a hepatotoxic agent as being the most important causal factor. We are in agreement with Oettlé's (1965) observation that " the association of hepatocellular carcinoma in Africa with the hyperplasia of post-necrotic cirrhosis, suggests that the cancers follow exposure to an agent producing necrosis followed by reactive hyperplasia ". In the context of this hypothesis, two factors deserve close study in Ethiopia, namely, mycotoxins and indigenous drugs.

Experimental studies have shown that mycotoxins are capable of producing liver necrosis, cirrhosis and hepatoma. Oettlé (1965) has presented evidence to indicate that a high incidence of primary carcinoma is associated with a greater mouldiness of food stuffs. He maintains that areas of high liver cancer incidence in Africa are all areas of high humidity. A high humidity is necessary for mould growth. Long storage of grains under unsatisfactory conditions entailing exposure to moisture supports mould growth and mycotoxin contamination. Coady (1965) examined random samples of food grains collected from Addis Ababa's Mercato, the largest grain market in Ethiopia. He isolated toxic aspergilli and *Penicillium islandicum* from various grains including " Teff ". (Teff, *Eragrostis abyssinica*, is a small grain used to prepare the national bread " ingerra " which is the staple diet in most areas of the country.) A significant amount of aflatoxin was found in sorghum, wheat and teff collected from different provincial markets in Ethiopia. It is therefore probable that mycotoxins play an important role in the causation of post-necrotic cirrhosis and primary carcinoma of the liver.

Like in many other African countries, indigenous medical practice is widely prevalent in Ethiopia. The exact herbs used in local remedies are not known in most instances. Schoental and Coady (1968) tested 40 different Ethiopian plants featuring in traditional cures, for hepatotoxicity. They found 10 species belonging to the genera Crotalaria, Cynoglossum, Heliotropium and Senecio, toxic to the liver of rats. Due to the prevalent practice of eating raw beef in Ethiopia, intestinal taeniasis is extremely common. A number of indigenous drugs in erratic doses are taken, in an attempt to eradicate the tape worm. Pankhurst (1968) lists 23 indigenous drugs as traditional taenicides of Ethiopia. The most popular of these drugs known as " Kosso " (the same word is used in Ethiopia for the parasite also) is the flower of the tree *Brayera anthelminthica* or *Hagenia abyssinica*. The usual dose is an infusion in " talla " (the local beer) of a handful of dried flowers. Sometimes a mixture of two taenicides is administered. It is customary to take a dose of taenicide each month or once in 2 months. In our series all the patients gave histories of taking " Kosso ", or one of these indigenous taenicides, regularly and the majority of them had taken the drug once in 2 months, or even at shorter intervals, for many years. These drugs are known to cause severe ill effects. Abortions caused by the administration of these drugs are frequently encountered. Several children who developed hepatitis and nephritis after administration of indigenous taenicides have been treated at the Ethio-Swedish Pediatric Clinic at Addis Ababa (Habte, personal communication). Jaundice is a common disorder and is invariably labelled as viral hepatitis. How many of these are the result of toxic injury to the liver is a question still to be resolved. A very careful and detailed study of these indigenous drugs is necessary to determine their hepatotoxicity. Circumstantial evidence is in favour of these being an important cause of liver injury in this country.

### SUMMARY

The clinico-pathological features of 38 cases of primary liver carcinoma proved at post-mortem examination are presented. These cases were seen during a 3-year period from January 1966 to December 1968 among 2880 adult patients admitted to the Department of Medicine of the Imperial Ethiopian Armed Forces Hospital at Addis Ababa. Primary liver carcinoma accounted for 20% of the total mortality in the medical wards of this hospital.

The majority of the patients (79%) were between the ages of 41 and 60 years. Abdominal pain was the constant symptom in all cases. The average duration of the disease since symptoms appeared to death was about 5 months.

Cirrhosis of the liver was present in 36 (94·7%) of the cases. Out of those with cirrhosis 27 (75%) were of the post-necrotic type. None of the livers showed fatty change, nutritional cirrhosis or parasites.

The possible aetiological factors are discussed. Mycotoxins and hepatotoxic indigenous drugs could play an important role in the causation of liver injury and primary liver carcinoma in Ethiopia.

### REFERENCES

ALPERT, M. E., HUTT, M. S. R. AND DAVIDSON, C. S.—(1968) *Lancet*, i, 1265.
BERMAN, C.—(1951) ' Primary carcinoma of the liver '. London (H. K. Lewis).
BLACHOS, J. AND KUBASTOVA, B.—(1963) *Ethiopian med. J.*, **1**, 190.
BURKITT, D. and SLAVIN, G.—(1968) ' Patterns of Cancer Distribution in Tanzania '. In ' Cancer in Africa .' Nairobi (East African Publishing House), p. 13.
COADY, A.—(1965) *Ethiopian med. J.*, **3**, 173, 177.
ESHELMAN, J. L.—(1966) *E. Afr. med. J.*, **43**, 273.
GALL, E. A.—(1960) *Am. J. Path.*, **36**, 241.
LINSELL, C. A.—(1968) ' Cancer in Kenya '. In ' Cancer in Africa '. Nairobi (East African Publishing House), p. 6.
MOLINEAUX, L., PLORDE, J. AND DOSNOY, J.—(1966) *Ethiopian med. J.*, **5**, 47.
OETTLÉ, A. G.—(1965) *S. Afr. med. J.*, **39**, 817.
PANKHURST, R.—(1968) *History Journal* (of the History Society of the Haile Selassie I University), **2**, 6.
PRATES, M. D.—(1958) *Br. J. Cancer*, **12**, 177.
SCHOENTAL, R. AND COADY, A.—(1968) *E. Afr. med. J.*, **45**, 577.
TEFERRA, A. AND ABDUL KADIR, J.—(1968) *Ethiopian med. J.*, **6**, 95.